# Assessment of Energy Intake and Energy Expenditure of Male Adolescent Academy-Level Soccer Players during a Competitive Week

**DOI:** 10.3390/nu7105400

**Published:** 2015-10-02

**Authors:** Marc A. Briggs, Emma Cockburn, Penny L. S. Rumbold, Glen Rae, Emma J. Stevenson, Mark Russell

**Affiliations:** 1 Department of Sport, Exercise and Rehabilitation, Faculty of Health and Life Sciences, Northumbria University, Newcastle upon Tyne NE1 8ST, UK; penny.rumbold@northumbria.ac.uk (P.L.S.R.); e.stevenson@northumbria.ac.uk (E.J.S.); mark.russell@northumbria.ac.uk (M.R.); 2 London Sport Institute, Middlesex University, London NW4 4BT, UK; e.cockburn@mdx.ac.uk; 3 MSK CATS, South Tyneside Foundation NE34 0PL, UK; glen.rae@stft.nhs.uk

**Keywords:** energy intake, energy expenditure, soccer, adolescent, male

## Abstract

This study investigated the energy intake and expenditure of professional adolescent academy-level soccer players during a competitive week. Over a seven day period that included four training days, two rest days and a match day, energy intake (self-reported weighed food diary and 24-h recall) and expenditure (tri-axial accelerometry) were recorded in 10 male players from a professional English Premier League club. The mean macronutrient composition of the dietary intake was 318 ± 24 g·day^−1^ (5.6 ± 0.4 g·kg^−1^ BM) carbohydrate, 86 ± 10 g·day^−1^ (1.5 ± 0.2 g·kg^−1^ BM) protein and 70 ± 7 g·day^−1^ (1.2 ± 0.1 g·kg^−1^ BM) fats, representing 55% ± 3%, 16% ± 1%, and 29% ± 2% of mean daily energy intake respectively. A mean daily energy deficit of −1302 ± 1662 kJ (*p* = 0.035) was observed between energy intake (9395 ± 1344 kJ) and energy expenditure (10679 ± 1026 kJ). Match days (−2278 ± 2307 kJ, *p* = 0.012) and heavy training days (−2114 ± 2257 kJ, *p* = 0.016) elicited the greatest deficits between intake and expenditure. In conclusion, the mean daily energy intake of professional adolescent academy-level soccer players was lower than the energy expended during a competitive week. The magnitudes of these deficits were greatest on match and heavy training days. These findings may have both short and long term implications on the performance and physical development of adolescent soccer players.

## 1. Introduction

Soccer is typically classified as a high-intensity, intermittent team sport comprised of two 45 min halves [1]. During a 90 min match, distances of 7–9 km have been reported for adolescents representing professional soccer academies [2,3,4]. In adult players of similar standard, match distances typically range between 9 and 13 km [5,6,7]. Similar match distances may insinuate comparable workloads between youth and adult populations. However, utilizing adult data to predict adolescent athletes’ energy expenditure has been criticized and may not be directly comparable, due to the increased energy cost of exercise [8]. Energy balance is integral for adolescents to sustain optimal growth and development [9,10], with additional nutritional intake required to offset the increased energy cost of high-level training and competition [11].

Research investigating soccer-specific nutritional intakes has been carried out in adult professional soccer [12,13,14,15,16,17]. However, a relatively limited amount of studies have investigated the nutritional intake of adolescent academy-level soccer players [18,19,20,21,22]. The findings of these studies, which have primarily investigated the habits of players from outside of the UK, have typically reported sub-optimal energy intake relative to estimates of energy expenditure [18,20,21,22]. In the only study to date to investigate dietary and activity regimes of adolescent soccer players in the UK, it was reported that dietary practices are inadequate to sustain the demands of training and competition, with a mean daily energy deficit of −3299 ± 729 kJ reported [23]. Although energy expenditure estimations utilized field-based methods, these equations were dependent upon subjective accounts of activity volume, recorded in self-reported training diaries. [18,20,21,22,23].

Assessing energy balance is reliant on the comparison of valid energy intakes against accurate methods of energy expenditure. Although doubly labelled water is deemed the gold standard when assessing energy expenditure [24], its complex approach may not be feasible for field-based researchers and practitioners. An alternative to this approach may be to adopt accelerometers, which have been demonstrated to elicit valid and reliable measures of physical activity in youth within free-living environments [25]. There are currently no studies, which have utilized accelerometry to assess energy expenditure, whilst also accounting for the potential under-reporting error of energy intake methods, within a full training and competition week in male adolescent Premier League academy-level soccer players in the UK.

Academy-level soccer players in the UK engage in mandatory training volumes of up to 20 h per week in accordance with the newly adopted Elite Player Performance Plan (EPPP) [26], with additional demands (school P.E.; county and/or national representation and other sporting commitments) independent of academy training, comprising training loads which are not comparable to full-time scholar soccer players. In order to better understand the dietary and activity habits of this population and to distinguish whether or not current dietary practices are adequate to meet the demands of training and match play and also growth and development, the aim of this study was to assess energy balance in male adolescent, academy-level soccer players over a seven day period that included four training days, one match day and two rest days. Based on previous studies, it was hypothesized that energy intake would be significantly less than energy expenditure.

## 2. Experimental Section

### 2.1. Participants

Ten male players (age: 15.4 ± 0.3 years; stature: 1.70 ± 0.06 m; body mass: 57.8 ± 7.8 kg and Body Mass Index: 19.84 ± 1.58 kg·m^−2^) who played for a Premier League soccer academy participated in the study. All participants were actively engaged in full training and competition, which over the course of the study consisted of four training days (two training sessions per day was classified as a heavy day and one session per day was classified as a moderate day), a match day and two non-training recovery days within a seven day period. Data collection period was during the second half of the competitive season (March), whereby participants were consistently engaged in 20 h of active training per week. Written informed consent was gained from both participants and their respective parents or guardians, once ethical approval was granted by the Faculty of Health and Life Sciences Research Ethics Committee at Northumbria University (approval number RE12-03-131775, 28th March 2013).

### 2.2. Dietary Assessment

Energy intake was recorded over a seven day period during the competitive season, using the combined method of self-reported weighed food diary, supplemented with 24-h recall [27]. Previous findings have demonstrated this method to be a valid alternative to current energy intake collection methods, when adjustment is made to offset under-reporting as previously described by Briggs *et al.* [27]. Seven day dietary data collection was seen as optimal to gain a sufficient amount of information whilst maintaining high compliance [28], and is indicative of previous studies [18,22,23].

Each participant was provided with a food diary and was asked to detail their weighed food intake, time of food consumption, preparation and cooking methods, and brand names. Provision of electronic portable scales facilitated weighing of all food items consumed. Prior to data collection, a series of practical workshops were delivered to all participants by the lead author in order to ensure that participants were familiar with the study procedures relating to energy intake. To coincide with the self-reported food diary each participant engaged in a 24-h recall interview on each day of the data collection period. Interviews were carried out using the two-pass method [29] whereby the overall eating events of the previous 24-h were reviewed to identify the main foods and beverages consumed; Secondly, the participants were prompted for more information such as condiments, brand names, additional food or drink items, how the foods were prepared and cooked and portion sizes if not provided in the food diaries.

Food diaries were cross-referenced with the respective 24-h recalls, supplementing any missing or additional information. Commercially available software was used to analyze energy intake (Microdiet version 2.8.5, Downlee Systems Limited, High Peak, UK). To ensure consistency, a single researcher, who was responsible for delivering the data collection familiarization workshops to the participants, performed all dietary analysis, as recommended by Deakin [30]. Total energy intake values were adjusted to accommodate for an under-reporting bias using a correction factor (Equation (1)) as previously identified by Briggs *et al.* [27].

(1) Energy Intake: *y* = 1.0397*x* – 0.1064 (where *y* = adjusted energy intake and *x* = participants self-reported energy intake).

### 2.3. Energy Expenditure Assessment

Energy expenditure was calculated using accelerometry methods (ActiGraph GT3X+; ActiGraph, Pensacola, FL, USA) that demonstrate valid and reliable measures of physical activity and sedentary time in youth populations [25]. This accelerometer demonstrates high levels of inter-instrument reliability (ICC values: 0.97 to 1.00 for raw outputs and 0.97 to 0.99 for derived outputs, within free-living environments; [31]). The accelerometer was positioned above the anterior spine of the iliac crest in line with the anterior axillary line of the dominant hip as per the manufacturer’s recommendations. The acceleration output was digitized by a 12-bit analogue-to-digital convertor at a user specific rate of 30 Hz. Participants were instructed to wear the device for 24 h for each of the seven days except during exposure to water-based activities (e.g., swimming and bathing). MET intensity thresholds were adopted based on previous calibration studies [32,33,34] (Table 1). In addition cut points devised by Evenson *et al.* [32] were used as they exhibit significantly better accuracy than other published cut points [35] (Table 1).

**Table 1:** MET Intensity Threshold and Cut Points

	MET Intensity Thresholds	Cut Points
Sedentary Activity (SED)	<1.5 METs	≤100
Light Physical Activity (LPA)	≥1.5 and <4 METs	>100 and
Moderate Physical Activity (MPA)	≥4 and <6 METs	≥2296 and <4012
Vigorous physical Activity (VPA)	≥6 METs	≥4012

Relevant METs and cut points were inserted to the ActiLife 6 Data Analysis Software (ActiGraph, Pensacola, FL, USA) accordingly, prior to energy expenditure calculations. In many research designs displaying energy expenditure in relation to METs is appropriate, however when comparing with energy intake, data expressed as kJ is more relevant and directly comparable. To calculate energy expenditure in kJ, daily MET values were derived from the raw data accelerations and input in to a modified version of the equation devised by Ridley *et al.* [36] (Equation (2)), using Schofield’s [37] prediction equation to estimate adolescent RMR (Equation (3)).

(2) Energy Expenditure (kJ) = MET value × adolescent RMR (kJ·kg^−^^1^·min^−^^1^) × kg body weight × number of minutes activity performed

(3) Resting Metabolic Rate (RMR) = 17.686 × kg body weight + 658.2

### 2.4. Statistical Analysis

Dietary intake data was considered reliable at <20% when using the percentage of relative standard error (SEM ÷ Mean) [23]. Seven day means for total energy intake (kJ), total energy expenditure (kJ), energy deficit (kJ) and macronutrients (% total energy intake, g, g·kg^−1^) were determined. A paired samples *t*-test was used to analyze differences in mean seven day energy balance and also differences in energy balance for different types of training/recovery days (heavy, moderate, rest) and match day. A one way (within-participants factor: energy deficit) repeated measures analysis of variance (ANOVA) was used to examine if energy deficit differed between days (heavy, moderate, rest and match day). A separate one way (within-participants factor: macronutrient intake) repeated measures ANOVA was used to examine if carbohydrate, protein and fat differed between days (heavy, moderate, rest and match day). Mauchly’s test was consulted and Greenhouse-Geisser correction was applied if the assumption of sphericity was violated. Significant main effects were further investigated using multiple pairwise comparisons with Bonferroni confidence interval adjustment. All data are presented as mean ± SD, with level of significance set at *p* ≤ 0.05, using SPSS (Version 21; SPSS Inc., Chicago, IL, USA) for all analysis.

## 3. Results

Estimates of nutritional intake are considered reliable as relative standard error did not exceed 8% for any of the analyzed macronutrients.

### 3.1. Macronutrients

The mean daily macronutrient intakes, with a breakdown in relation to type of activity day are expressed in Table 2. The ANOVA revealed no significant main effect for carbohydrate (*F*(3,27) = 1.671, *p* = 0.20), protein (*F*(3,27) = 0.883, *p* = 0.46) and fat (*F*(3,27) = 1.963, *p* = 0.14).

**Table 2:** Mean macronutrient intakes of adolescent soccer player’s diets broken down by type of activity day (mean ± SD).

Macronutrient	Heavy	Moderate	Rest	Match	Mean
*Protein*					
Per day (g·day^−^^1^)	93 ± 29	82 ± 27	96 ± 22	86 ± 26	86 ± 10
Per unit BM (g·kg^−^^1^·day^−^^1^)	1.6 ± 0.5	1.4 ± 0.6	1.7 ± 0.5	1.5 ± 0.5	1.5 ± 0.2
Total protein energy ratio (%)	17 ± 6	16 ± 4	19 ± 6	17 ± 4	16 ± 1
*Carbohydrate*					
Per day (g·day^−^^1^)	337 ± 109	321 ± 76	281 ± 51	314 ± 97	318 ± 24
Per unit BM (g·kg^−^^1^·day^−^^1^)	6.0 ± 2.3	5.6 ± 1.6	5.0 ± 1.3	5.5 ± 2.0	5.6 ± 0.4
Of which are sugars (g·day^−^^1^)	155 ± 71	150 ± 53	103 ± 38	109 ± 56	136 ± 24
Total carbohydrate energy ratio (%)	55 ± 7	58 ± 8	49 ± 7	55 ± 8	55 ± 3
*Fats*					
Per day (g·day^−^^1^)	73 ± 24	66 ± 26	80 ± 19	66 ± 18	70 ± 7
Per unit BM (g·kg^−^^1^·day^−^^1^)	1.3 ± 0.4	1.1 ± 0.5	1.4 ± 0.3	1.1 ± .0.2	1.2 ± 0.1
Of which are saturates (g·day^−^^1^)	28 ± 11	26 ± 10	27 ± 7	21 ± 8	26 ± 3
Total fats energy ratio (%)	28 ± 6	27 ± 8	33 ± 6	28 ± 6	29 ± 2

### 3.2. Energy Balance

Mean daily energy intake (9395 ± 1344 kJ) was significantly lower than mean daily energy expenditure (10679 ± 1026 kJ) (*p* < 0.05). This resulted in a mean daily energy deficit of −1302 ± 1662 kJ.

### 3.3. Energy Cost of Activities

**Figure 1:**
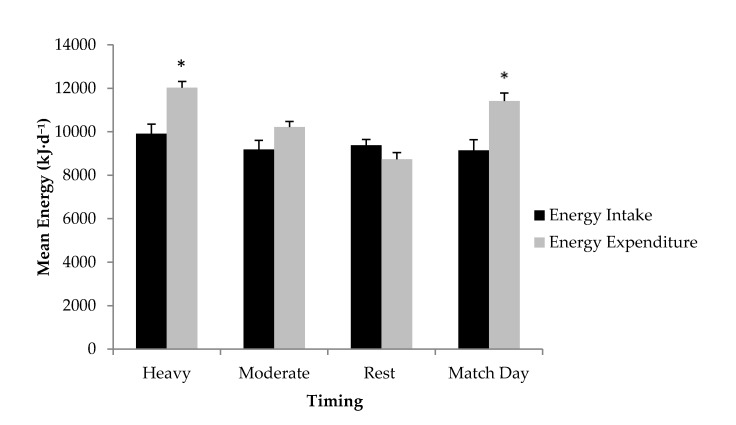
Mean Energy Intake (kJ) compared to Mean Energy Expenditure (kJ) for type of training. * Significant difference between mean energy intake and mean energy expenditure at the corresponding time-point at *p* < 0.05 level.

Figure 1 illustrates the mean daily energy intake and expenditure data based on the type of training day. A significant difference was observed between mean energy intake and energy expenditure on heavy training days (−2114 ± 2257 kJ) (*p* < 0.05) and match day (−2278 ± 2307 kJ) (*p* < 0.05). Although an energy deficit was also observed on a moderate training day (−1034 ± 2022 kJ) this was not statistically significant (*p* = 0.14). Rest day was the only exception which identified a mean positive energy balance (640 ± 1197 kJ); yet this this value was similar to energy expenditure (*p* = 0.125). The ratio of mean energy intake to mean energy expenditure was 89% ± 16%.

The ANOVA revealed a significant main effect (*F*(3,27) = 6.682, *p* < 0.01), with *post hoc* comparisons identifying significant differences between heavy training days and rest day (−2755 ± 745 kJ; *p* < 0.05), and also between a match day and rest day (−2918 ± 745 kJ; *p* < 0.01).

## 4. Discussion

The primary aim of the study was to assess energy balance in male adolescent academy-level soccer players. The findings demonstrate that over a seven day period players were in a negative energy balance, with energy intake being insufficient to meet the demands of training and competition. Mean daily energy intake was significantly lower than mean daily energy expenditure, providing a daily energy deficit. Additionally, type of training had a direct impact on the degree of energy deficit, highlighting that heavy training days and match days to be a particular threat to energy balance. Such information is likely of use to practitioners and players who should consider adjusting energy intake accordingly.

This is the first study to utilize accelerometry to assess energy expenditure, whilst accounting for energy intake during the same competitive week in adolescent academy-level soccer players competing in the UK. While magnitudes of deficits differ, evidence of negative energy balance reflects observations from similar populations competing outside of the UK [18,19,20,21,22]. In an attempt to quantify the magnitude of the mean energy deficit (−1302 ± 1662 kJ·day^−^^1^), the findings of the present study provided a considerably lower deficit when compared to previous authors (e.g., −3726 ± 3073 kJ·day^−1^ [18]; −3299 ± 729 kJ·day^−1^ [23]). However caution must be exercised when attempting to apply direct comparisons due to methodological differences between study designs [38] and the absence of post body mass measurement. The greater deficit in the previous studies may be inflated due to using an estimated method of measuring energy expenditure; 14,445 ± 1089 kJ·day^−1^ [18] and 15,148 ± 255 kJ·day^−1^ [23], in comparison to the current study’s findings (10,679 ± 1026 kJ·day^−1^), which used accelerometry as a more sensitive measure of energy expenditure.

To contextualize the energy deficit (−1302 ± 1662 kJ·day^−^^1^) in relation to weight loss, this would equate to a mean weekly weight loss of 0.04 ± 0.05 kg per player, using previously published equations [39]. Energy deficit coinciding with heavy training over a sustained period may cause detriments to health, impacting on optimal growth and development [11,40], in addition to performance detriments and increased risk of injury [41]. However due to external factors, participants were unavailable for body mass measurement during the immediate post-data collection period to determine if weight loss was evident, therefore energy deficit results should be interpreted with caution.

Information is lacking in the literature to determine where the greatest energy deficits are occurring throughout the week. Although studies outline mean energy expenditure values [18,19,20,21,22], these studies do not provide a breakdown of the difference between days. Within the current study a significant weekly deficit was observed, however only heavy training (−2114 ± 2257 kJ) (*p* < 0.05) and match day (−2278 ± 2307 kJ) (*p* < 0.05) induced a significant energy deficit relative to energy expenditure. The deficits identified may have implications as Wang *et al.* [42] proposed a difference in energy intake of 461–691 kJ·day^−1^ to be clinically meaningful in a weight loss context. However, this could not be confirmed due to the inability to measure post-data collection body mass. In addition macronutrient intake was not significantly different between days. Findings may suggest that macronutrient consumption and total energy intake is relatively stable across the week, therefore the issue is that players are not adjusting intake to account for the intensity of training. This finding supports previous research, albeit in Professional Rugby League, whereby players’ energy intake was not adjusted for type of training day, in addition, energy derived from carbohydrate remained stable throughout the week [43]. Recommendations of periodized nutritional intake may be advised to account for training intensity and volume [44], reflecting energy intake with the type of training day, either increasing or decreasing total consumption accordingly, to ensure a slight positive energy balance is achieved to not only optimize performance [11] but to sustain optimal growth and development [9,10].

Burke *et al.* [45] proposes 5–7 g·kg^−1^·day^−1^ for moderate training, increasing to 7–10 g·kg^−^^1^·day^−1^ for intensive training. The current study’s mean daily carbohydrate intake finding of 55% ± 3%, equating to 5.6 ± 0.4 g·kg^−1^·day^−^^1^, comprised mainly of starchy foods such as breads, cereals and pasta, demonstrate carbohydrate contribution to total daily energy intake to likely be sub-optimal, especially during heavy training sessions and match days (Table 2). This finding supports previous published research within this population with carbohydrate intake ranging from 45% to 56% [18,19,20,21,22,23,46]. Diets high in carbohydrate enable an increased muscle glycogen concentration, subsequently delaying the onset of fatigue and sustaining performance levels [47,48]. However, it is also acknowledged that training with low carbohydrate availability may augment adaptive responses to exercise training [49,50]. Soccer specific studies have identified optimal carbohydrate intake to improve total match distance [51] and ability to perform at high-intensity [52]. However, chronic periods of sub-optimal energy intake, derived from inadequate carbohydrate consumption, coinciding with sustain periods of high training volumes may impair growth and development and effect performance levels [11,40,41].

Proteins are essential in recovery and to support gains in lean mass [53] and maintenance following muscle damaging exercise [54]. In the limited amount of nitrogen balance studies in adolescent soccer players, a recommendation of 1.4–1.7 g·kg^−1^·day^−1^ [22], has been suggested which aligns with adult counterparts (1.2–1.7 g·kg^−1^·day^−^^1^) [53,55]. The current study’s findings of 1.5 ± 0.2 g·kg^−1^·day^−1^ proposes protein intake is within the recommended range to optimize recovery and development, which was consumed mostly from poultry and dairy foods. This finding is not surprising with previous research reporting young athletes demonstrate adequate protein intakes, despite being in negative energy balance [56]. Optimal protein intake provides essential amino acids to support growth and development of lean body mass [11]. However, whilst acknowledging adequate protein intakes may be achieved with sub-optional energy intake, caution is required, as protein may be used as a substrate for energy, impacting on the ability to synthesize lean tissue.

Younger athletes have a greater reliance on fat as a fuel source during exercise [57], however there is currently no data recommending adolescent athletes consume a higher fat intake than adults. Given the high-intensity nature of soccer, fat recommendations are based upon facilitating carbohydrate intake, as opposed to contribution for energy metabolism [15]. Soccer-specific research suggests a fat intake of <30% [15]. Studies assessing fat intake in male adolescent academy soccer players have reported intakes of 29%–38% [18,19,20,21,22,23,46], which are borderline or above recommendations. The current study’s mean fat intake (29% ± 2%) may explain the limited consumption of optimal levels of carbohydrate, due to fat intake approaching the top limit of recommended consumption. Furthermore the quality of fats is important to consider with recommendations of <10% of total energy intake derived from saturated fatty acids [38]. However, the current study found saturated fat values of 26% ± 3%. Considering the significant energy deficit within the current sample of players as well as recognizing recent research reporting utilization of fat as a fuel source to spare glycogen depletion [49,50], albeit equivocal, a reduction in fat intake may not be advisable. Moreover a reduction in saturated fatty acids may provide opportunity for consumption of unsaturated fats or higher intakes of carbohydrate, especially on match day to sustain performance levels.

It is acknowledged that all methods of measuring dietary intake have inherent limitations and may be affected by errors of precision and validity [58]. However, the current study is the first to adopt the combined method of a self-report weighed food diary, supplemented with daily 24-h recall, which has previously been validated in male adolescent academy-level soccer players [27] and other adolescent populations engaged in regular exercise [59]. The current study utilized the combined method and applied the correction equation to adjust energy intake data to accommodate for a slight under-reporting bias, which has been previously established within this population [27]. The current study also represents high ecological validity through the use of a free-living experimental design. Previous studies which have assessed energy intake and expenditure within formalized training centers [21,46] may increase internal validity exerting greater control, but exclude influences of habitual family and school environment. Furthermore, whilst it is accepted that the data collection period may only provide a limited insight in to a 9 month long competitive season, a seven day energy intake collection period used within the current study is seen as optimal to increase reliability and validity whilst minimizing the burden of longitudinal collection periods [28]. In addition, the incorporation of the EPPP [26] stipulating compulsory training volumes of 20 h per week for academy soccer players ensure that there is little differentiation in training volume within the competitive periodization phase.

Measures of energy expenditure are diverse in the literature [18,19,20,21,22,23,46], with some studies failing to produce a quantification of energy cost whilst attempting to provide conclusions of optimal dietary practices [19]. The inconsistent approaches provide difficulties in accurately comparing the energy demands placed upon adolescent academy soccer players. Techniques such as doubly-labelled water and indirect calorimetry, although accurate [24], are complex, expensive and not applicable to a habitual setting. The current study utilized accelerometers, which have been demonstrated to elicit valid and reliable measures of physical activity and sedentary time in youth [25], providing an objective quantification of energy expenditure within a free-living environment. However, whilst it is accepted that energy expenditure measured by accelerometry produces high correlation with indirect calorimetry [31,60], there is no absolute agreement, therefore limitations do need to be acknowledged when adopting such methods.

## 5. Conclusions

In conclusion, over a seven day period male adolescent academy-level soccer players were in a negative energy balance. This may have longer term implications impacting on the ability to sustain the demands of training and competition as well as maintaining optimal growth and development. In particular, heavy training and match days are of concern, with players not adjusting energy intake to combat the increased energy cost. Findings support previous research from outside of the UK, although demonstrating a lower, yet significant, mean daily energy deficit. Further interventions and education on nutritional strategies for academy soccer players should be considered relative to training and match play requirements to prevent detriments to optimal growth, development and performance.
